# The Relationship between Physical Activity and Health-Related Quality of Life in Korean Adults: The Eighth Korea National Health and Nutrition Examination Survey

**DOI:** 10.3390/healthcare11212861

**Published:** 2023-10-30

**Authors:** Mun-Gyu Jun, Se-Hyeon Han

**Affiliations:** 1 Department of Coaching, College of Physical Education, Kyung Hee University (Global Campus), Yongin 17014, Republic of Korea; mkrollcage@khu.ac.kr; 2 School of Media Communication, Hanyang University, Seoul 04763, Republic of Korea

**Keywords:** health, nutrition surveys, exercise, quality of life, health policy

## Abstract

This study used the raw data from the Eighth Korea National Health and Nutrition Examination Survey (KNHANES-VIII), conducted under the supervision of the Ministry of Health and Welfare and the Korea Centers for Disease Control and Prevention in 2019. It was conducted to identify a significant correlation between physical activity (PA) and health-related quality of life (HR QOL) in the Korean population. In 2019, the KNHANES-VIII added the Health-related Quality of Life Instrument with 8 items (HINT-8) to assess the HR QOL. The independent variable is related to PA, specifically the presence or absence of PA, type of PA, and the frequency of PA. The dependent variable is HR QOL, measured either as the total score or specific items (e.g., pain, vitality, and memory) using the HINT-8 measurement tool. Demographic characteristics and health status may directly or indirectly influence the relationship between PA and HR QOL, which were used as covariates. A total of 4357 participants were included in the current study. The mean HINT-8 scores were significantly higher in the participants who performed PA on a weekly basis, leisure-related PA or MSPA, as compared with those who did not (*p* = 0.01 and <0.0001, respectively). In both the unadjusted and adjusted models, the mean HINT-8 scores were significantly higher in the participants who performed ≥500 MET-min/week of leisure-related PA as compared with those who did not (95% CI: 1.017–1.033; *p* < 0.001 and 95% CI: 1.005–1.02; *p* = 0.001, respectively). In both the unadjusted and adjusted models, the mean HINT-8 scores were significantly higher (95% CI: 1.015–1.03; *p* < 0.001 and 95% CI: 1.004–1.018; *p* = 0.003, respectively) in the participants who performed MSPA for ≥2 days/week as compared with those who did not. The current results confirmed that there is a significant positive correlation between the PA and HR QOL based on the HINT-8 scores. Because the HINT-8 was developed to assess the HR QOL in Koreans, however, further studies are warranted to evaluate its applicability to other ethnic populations.

## 1. Introduction

According to the World Health Organization (WHO), health entails both a lack of illness and the presence of mental and social well-being [1]. The WHO also defines the quality of life (QOL) as “individuals’ perception of their position in life in the context of their culture and value systems in which they live, and in relation to their goals, expectations, standards and concerns”. QOL is a broad concept that is affected in diverse ways by an individual’s physical health, mental state, level of independent living, social relations, and relationship with the environment [2]. Based on the direct relationship of QOL with an individual’s happiness and achievement of life goals, it is considered as an essential factor for identifying success in the life of an individual. Of these, health-related QOL (HR QOL) serves as an indicator of health and well-being that has a direct impact on an individual’s physical, psychological, social, and mental health [3].

To date, physical activity (PA) has been suggested as a factor that is closely associated with both improved QOL and well-being [1]. Mechanisms underlying the potential relationship between PA and QOL/well-being include PA-induced changes in brain neurotransmitters and endogenous opioids that are involved in depression, anxiety, and other mood constructs [4,5]. It has also been reported that a latent conceptualization of psychological distress, such as depression, anxiety, stress, and sleep disturbance, had a significant correlation with improvements in well-being/satisfaction with life [6].

PA promotion is currently considered as a key component of public health policy. The effects of PA on health status have been mostly studied using objective outcome measures including HR QOL, serving as an indicator of individuals’ perception of their own health. Thus, the relationship between PA and HR QOL has been studied in the context of the effects of an exercise training program on HR QOL [7,8,9]. To date, such a relationship has been explored under the cross-sectional design [10,11,12,13,14,15].

Given the above background, this cross-sectional study used the raw data from the Eighth Korea National Health and Nutrition Examination Survey (KNHANES-VIII), conducted under the supervision of the Ministry of Health and Welfare (MHW) and the Korea Disease Control and Prevention Agency (KDCA) in 2019. In 2019, the KNHANES-VIII added the Health-related Quality of Life Instrument with 8 items (HINT-8) to assess the HR QOL. It was therefore conducted to identify a significant correlation between PA and HR QOL in the Korean population.

## 2. Materials and Methods

### 2.1. Study Setting and Participants

The Korean National Health and Nutrition Examination Survey (KNHANES) is a program to monitor the health behavior of individuals, the prevalence of chronic diseases, and the actual state of food and nutritional intake; it is conducted in accordance with Article 16 of the National Health Promotion Act, which involves government-designated statistics (Approval No. 117002) based on Article 17 of the Statistics Act. The KNHANES data are provided by the KDCA in compliance with the Personal Information Protection Act and Statistics Act. Moreover, the KDCA obtains written informed consent from the participants.

The KNHANES was performed every 3 years between 1998 and 2008. Since 2008, however, it has been performed every year.

### 2.2. Study Design

The current study controlled factors that may influence participants’ HR QOL, such as sex, age group, level of education, monthly household income, marital status, smoking, alcohol intake, BMI, and medical history.

### 2.3. Measurement of Variables

The independent variable in the current study is related to PA, specifically the presence or absence of PA, type of PA (e.g., leisure-related PA or muscle-strengthening PA (MSPA)), and the frequency of PA (e.g., ≥twice/week). The dependent variable is HR QOL, measured either as the total score or specific items (e.g., pain, vitality, and memory) using the HINT-8 measurement tool. Demographic characteristics and health status may directly or indirectly influence the relationship between PA and HR QOL, which were used as covariates in the current study.

### 2.4. Characteristics of PA

Data were obtained from the KNHANES website and the Korean version of the Global Physical Activity Questionnaire (GPAQ) was used. The GPAQ comprises questions about performing PAs during the week. It was created by the WHO and is a standardized tool used to measure the levels of PAs, such as work, leisure, and travel.

The independent variable of PA in the current study was evaluated based on responses to the questions in the KNHANES-VIII. The survey included questions on the presence or absence of PA (e.g., participation or non-participation in PA), type of PA (e.g., work-related or leisure-related PA), and the intensity of PA (e.g., moderate-to-high-intensity PA). The participation in PA over the past 7 days was determined through a question, and the type of PA, mean daily duration, and weekly participation frequency were used to assess the pattern of PA.

Moderate-intensity PA refers to activities that cause slight shortness of breath or a slightly elevated heart rate, while high-intensity PA refers to vigorous activities that cause significant shortness of breath or a very fast heart rate. In the current study, moderate-intensity and high-intensity PAs were separately assessed for work-related and leisure-related ones. Some specific examples include work-related moderate-intensity PAs such as brisk walking (during work), light lifting or carrying objects, cleaning, and childcare activities (bathing, carrying a child), while work-related high-intensity ones include heavy lifting or carrying heavy objects (over 20 kg), digging, labor-intensive work on construction sites, and carrying objects up stairs. Leisure-related moderate-intensity PAs include brisk walking, light jogging, weight training (strength training), golf, dance sports, and Pilates, while leisure-related high-intensity ones include running, jumping rope, hiking, basketball matches, swimming, badminton, and so on.

The level of leisure-related PA was quantified using the weekly amount in MET-min/week, based on the metabolic equivalent of task (MET) index, as previously described [16]. That is, (1) moderate-intensity PA = 4.0 MET × daily duration of moderate-intensity PA (minutes) × weekly frequency of moderate-intensity PA; (2) high-intensity PA = 8.0 MET × daily duration of high-intensity PA (minutes) × weekly frequency of high-intensity PA; (3) total moderate-to-high-intensity PA = moderate-intensity PA + high-intensity PA. The global health recommendation for PA states that achieving 150 min or more of moderate-intensity exercise per week or 75 min or more of high-intensity exercise per week corresponds to 500 MET-min/week [17]. Therefore, a threshold of 500 MET-min/week was used as a criterion for distinguishing between the participants performing leisure-related PA according to the global health recommendation.

### 2.5. Measurement of HR QOL

HR QOL was measured using the HINT-8 assessment tool. The HINT-8 tool consists of four domains and eight items [18]. The items are rated using a 4-point Likert scale (1 = always good; 2 = usually good; 3 = sometimes good; 4 = poor). Lower scores indicate better HR QOL (1 = I am always happy; 2 = I am usually happy; 3 = I am sometimes happy; and 4 = I am never happy). The domains, items, and levels of responses that make up the HINT-8 are shown in Table 1.

In the current study, the participants’ responses to the HINT-8 items were used to calculate the HINT-8 total scores, as previously described [19]. The HINT-8 total score ranges from 0 to 1, where a score closer to 1 indicates higher HR QOL.

### 2.6. Assessment of Demographic Characteristics and Health Behavior of the Participants

For the current study, the participants were divided into age groups: 19–34 years old, 35–49 years old, and 50–64 years old. Levels of education were classified based on the final educational attainment; these include elementary school graduates, middle school graduates, high school graduates, and ≥college or university graduates. Monthly household income was divided into four quartiles (low, middle low, middle high, and high). Marital status was categorized as married for those who were married (including cohabiting or separated), and others for those who were widowed, divorced, or did not respond. Alcohol intake was based on the frequency of alcohol consumption in the past year, categorized as “abstinent” for those who did not drink at all, “low-moderate” for those who drank less than once a month or about once a month, “occasional” for those who drank 2–4 times a month or 2–3 times a week, and “frequent” for those who drank more than 4 times a week. Smoking status was classified as “current smoker” for those who responded “smoke every day” or “smoke occasionally”, “former smoker” for those who responded “used to smoke but not currently”, and “non-smoker” for those who responded “never smoked”. BMI was categorized as normal (BMI < 23 kg/m^2^), overweight (23–25 kg/m^2^), and obese (BMI ≥ 25 kg/m^2^) according to the 2018 Korean Society for the Study of Obesity Guidelines [20]. The presence of chronic diseases was determined based on the “disease” section of the KNHANES-VIII, including cancer (e.g., stomach, liver, colorectal, breast, cervical, lung, and thyroid cancer), vascular diseases (e.g., hypertension, dyslipidemia, stroke, myocardial infarction, and angina), joint diseases (e.g., osteoarthritis, rheumatoid arthritis, and osteoporosis), metabolic diseases (e.g., diabetes and thyroid diseases), and other diseases (e.g., pulmonary tuberculosis and asthma). The participants who received a diagnosis from a physician for any of these diseases were classified as having the respective disease, while those who did not receive a diagnosis were classified as the non-disease group.

### 2.7. Data Analysis

The publicly available data were obtained and then cross-checked to assess for completeness by matching the computed data with the available responses to the questionnaires, checking and setting the missing values based on reasonably possible ranges and skimming through the frequencies of each study variable to ensure that the distribution considered outliers [21]. All statistical analyses were performed using SPSS version 25.0 for windows (SPSS Inc., Chicago, IL, USA). Data were expressed as the number of the participants, mean ± standard deviation, or mean ± standard error, where appropriate. Both Mann–Whitney *U*-test and analysis of covariance (ANCOVA) were performed to analyze differences in the HINT-8 scores depending on the pattern of PA, for which demographic and socio-economic characteristics, health behaviors, and the underlying presence of chronic illness served as adjustment variables. A post hoc analysis was also performed using Bonferroni correction. A logistic regression analysis was also performed to identify a causal relationship between PA and the HINT-8 scores, for which both the unadjusted and adjusted models were used and odds ratio (OR) and 95% confidence intervals (CIs) were provided. A *p*-value of <0.05 was considered statistically significant.

## 3. Results

### 3.1. Demographic Characteristics of the Participants

**Figure 1:**
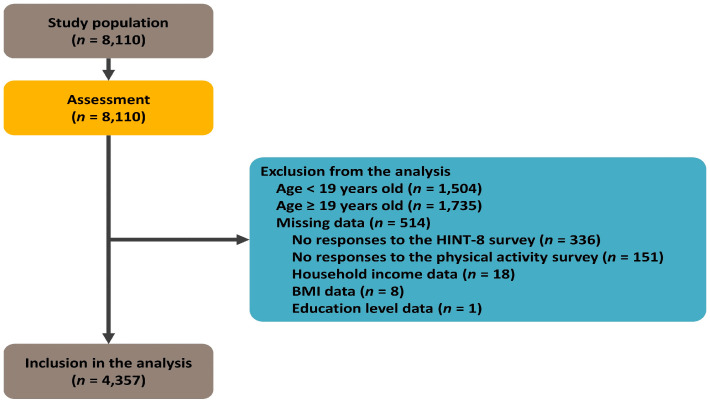
The study flowchart.

The raw data of the Eighth KNHANES included 4800 households within 192 survey areas nationwide, and it encompassed data from 8110 individuals aged ≥1 year old. Initially, 1504 minors under the age of 19 and 1735 elderly individuals aged ≥65 years old were excluded, resulting in a sample of 5102 participants. Subsequently, 151 individuals with incomplete or non-responsive data regarding exercise-related characteristics and 336 individuals with incomplete or non-responsive data for the HINT-8 items were further excluded, resulting in a sample of 4618 participants. Finally, 27 participants who did not respond to demographic questionnaire items on education level (*n* = 1), income level (*n* = 18), and body mass index (BMI)-related questions (*n* = 8) were excluded, and a total of 4357 participants were selected as the final study sample. The flowchart of the participant selection process is presented in Figure 1.

A total of 4357 participants were included in the current study; whose median age was 45 (range: 35–55) years old. Demographic characteristics of the participants are represented in Table 2.

### 3.2. Differences in the HINT-8 Scores Depending on the Pattern of PA

**Figure 2:**
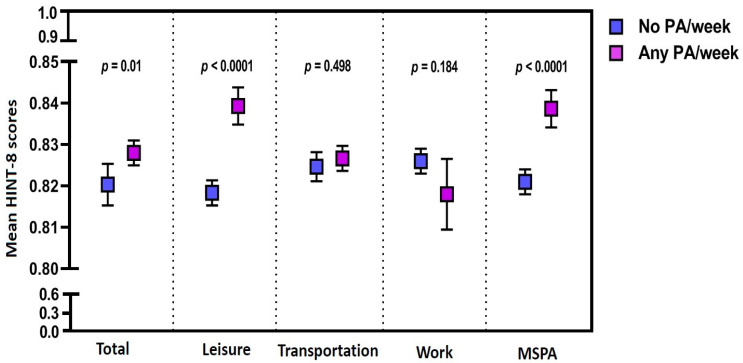
The Health-Related Quality of Life Instrument with 8 Items scores depending on the pattern of physical activity. Abbreviations: HINT-8, the Health-Related Quality of Life Instrument with 8 Items scores; PA, physical activity; MSPA, muscle-strengthening physical activity.

The mean HINT-8 scores were significantly higher in the participants who performed PA on a weekly basis as compared with those who did not (*p* = 0.01). Moreover, the mean HINT-8 scores were also significantly higher in the participants who performed leisure-related PA or MSPA as compared with those who did not (*p* < 0.0001) (Figure 2).

### 3.3. Relationship between Leisure-Time PA and the HINT-8 Scores

In the unadjusted model, the HINT-8 scores were significantly higher, by 2.510%, in the participants who performed ≥500 MET-min/week of leisure-related PA as compared with those who did not (95% CI: 1.017–1.033; *p* < 0.001) (Table 3).

In the adjusted model, the HINT-8 scores were significantly higher, by 1.27%, in the participants who performed ≥500 MET-min/week of leisure-related PA as compared with those who did not (95% CI: 1.005–1.02; *p* = 0.001). By levels of education, the HINT-8 scores were significantly higher—by 1.67% (95% CI: 1–1.033; *p* = 0.046) in the middle school graduates, by 4.37% (95% CI: 1.03–1.058; *p* < 0.001) in the high school graduates, and by 4.56% (95% CI: 1.031–1.06; *p* < 0.001) in the ≥college or university graduates—as compared with the elementary school graduates. By monthly household income, the HINT-8 scores were significantly higher—by 2.37% (95% CI: 1.012–1.035; *p* < 0.001) in the participants with mid-low monthly household income, by 3.59% (95% CI: 1.024–1.047; *p* < 0.001) in the participants with mid-high monthly household income, and by 3.53% (95% CI: 1.024–1.047; *p* < 0.001) in the participants with high monthly household income—as compared with the participants with low monthly household income. By the frequency of alcohol intake, the HINT-8 scores were significantly higher, by 0.91% (95% CI: 1–1.018; *p* = 0.047), in the participants with occasional alcohol intake as compared with those with abstinent alcohol intake. By sex, the HINT-8 scores were significantly lower, by 3.82% (95% CI: 0.954–0.969; *p* < 0.001), in women as compared with men. By age group, the HINT-8 scores were significantly lower—by 1.41% (95% CI: 0.977–0.995; *p* = 0.003) in the participants aged between 35 and 49 years old and by 2.22% (95% CI: 0.968–0.988; *p* < 0.001) in the participants aged between 50 and 64 years old—as compared with those aged between 19 and 34 years old. By the BMI, the HINT-8 scores were significantly lower, by 0.93% (95% CI: 0.984–0.998; *p* = 0.007), in the participants with BMI > 25 kg/m^2^ as compared with those with BMI < 25 kg/m^2^. By the medical history, the HINT-8 scores were significantly lower—by 1.71% (95% CI: 0.974–0.992; *p* < 0.001) in the participants with dyslipidemia and by 4.81% (95% CI: 0.924–0.981; *p* = 0.001) in those with cerebrovascular diseases—as compared with those with no medical history (Table 4).

### 3.4. Relationship between MSPA and the HINT-8 Scores

In the unadjusted model, the HINT-8 scores were significantly higher by 2.26% (95% CI: 1.015–1.03; *p* < 0.001) in the participants who performed MSPA for ≥2 days/week as compared with those who did not (Table 5).

In the adjusted model, the HINT-8 scores were significantly higher by 1.06% (95% CI: 1.004–1.018; *p* = 0.003) in the participants who performed MSPA for ≥2 days/week as compared with those who did not. By levels of education, the HINT-8 scores were significantly higher by 4.32% (95% CI: 1.029–1.058; *p* < 0.001) in the high school graduates and 4.51% (95% CI: 1.03–1.06; *p* < 0.001) in the ≥college or university graduates as compared with the elementary school graduates. By monthly household income, the HINT-8 scores were significantly higher by 2.35% (95% CI: 1.012–1.035; *p* < 0.001) in the participants with mid-low monthly household income, 3.61% (95% CI: 1.025–1.048; *p* < 0.001) in those with mid-high monthly household income and 3.58% (95% CI: 1.024–1.047; *p* < 0.001) in those with high monthly household income as compared with those with low monthly household income. By the frequency of alcohol intake, the HINT-8 scores were significantly higher by 0.93% (95% CI: 1–1.018; *p* < 0.042) in the participants with occasional alcohol intake as compared with those with abstinent alcohol intake. By sex, the HINT-8 scores were significantly lower by 3.79% (95% CI: 0.955–0.97; *p* < 0.001) in women as compared with men. By age, the HINT-8 scores were significantly lower by 1.40% (95% CI: 0.977–0.995; *p* = 0.003) in the participants aged between 35 and 49 years old and 2.28% (95% CI: 0.967–0.987; *p* < 0.001) in those aged between 50 and 64 years old as compared with those aged between 19 and 34 years old. By economic activities, the HINT-8 scores were significantly lower by 1.56% (95% CI: 0.978–0.991; *p* < 0.001) in the participants with no economic activities as compared with those with them. By smoking status, the HINT-8 scores were significantly lower by 1.27% (95% CI: 0.979–0.996; *p* = 0.003) in the ex-smokers and 2.83% (95% CI: 0.963–0.981; *p* < 0.001) in the smokers as compared with non-smokers. By BMI, the HINT-8 scores were significantly lower by 0.88% (95% CI: 0.984–0.998; *p* = 0.011) in the participants with BMI > 25 kg/m^2^ as compared with those with BMI < 25 kg/m^2^. By medical history, the HINT-8 scores were significantly lower by 1.70% (95% CI: 0.974–0.992; *p* < 0.001) in the participants with dyslipidemia and 4.84% (95% CI: 0.924–0.98; *p* = 0.001) in those with cerebrovascular diseases as compared with those with no medical history (Table 6).

## 4. Discussion

In recent decades, there has been a substantial increase in the life expectancy of individuals, which has resulted in an increase in the number of those with chronic diseases and disabilities [22,23]. It would therefore be mandatory to promote behaviors that might prevent disability and hospitalization. In this context, PA has been established as a beneficial modality for the prevention and treatment of non-communicable diseases and other chronic conditions and in improving the HR QOL [10,24,25,26,27].

To date, the positive correlation between PA and HR QOL has been established. Active PA had a positive effect on both the physical and mental domains of HR QOL in patients with diabetes and the elderly [28,29,30]. According to a systematic review of previous research, there was a positive correlation between PA and HR QOL in the general adult population [10]. This is advocated by other studies showing that PA intervention was effective in significantly improving the QOL in clinical and healthy populations [31,32,33]. Published research has also shown that a higher level of PA in the work and home environment or during leisure time had a significant positive correlation with HR QOL irrespective of underlying conditions [34]. Moreover, Bădicu G. showed a significant positive correlation between PA and HR QOL irrespective of the type of exercise in adults [35]. Furthermore, Subramaniam M et al. also showed a positive correlation between the level of PA and HR QOL in individuals with comorbidities [36].

According to previous studies, HR QOL is closely associated with both sociodemographic and lifestyle factors; its domains include physical, emotional, and social functions [37,38,39,40,41]. It therefore serves as an essential public health instrument to assess the physical and social functioning, mental health, and well-being of an individual and to evaluate the effects of population-based intervention programs [42].

To date, many HR QOL questionnaires have been developed to assess the QOL in diverse settings [43,44,45,46]. Of these, the EuroQol five-dimension (EQ-5D) questionnaire has been used to assess QOL in the KNHANES studies before the KNHANES-VIII [3,47,48,49,50,51]. In 2019, however, the KDCA developed the HINT-8 to more accurately measure HR QOL in Koreans [52]. It consists of four domains (physical, social, mental, and positive) and eight details (stair climbing, pain, energy, working, depression, memory, sleep, and happiness). Each domain has four levels of questions. This makes it possible to express the level of health status more abundantly as compared with the EQ-5D. Moreover, it has a lower ceiling effect as compared with the EQ-5D [53]. For these reasons, the HINT-8 has been used as an HR QOL assessment tool for the KNHNES studies since 2019 [18,52,54,55].

According to the global recommendations on physical activity for health, adults and the elderly are recommended to perform PA of minimum moderate intensity (MPA), corresponding to 3–6 METs, for a total of 150 min/week, ≥75 min of vigorous-intensity PA (VPA) (≥6 METs), or any equivalent combination of those for ≥10 min duration [56]. The current study performed an analysis based on the PA guidelines for Koreans provided by the MHW and the global PA recommendations. It used criteria of 500 MET-min/week and ≥2 days/week for leisure-related PA and MSPA, respectively.

To summarize, the current results are as follows:

(1) The mean HINT-8 scores were significantly higher in the participants who performed PA on a weekly basis, leisure-related PA, or MSPA as compared with those who did not (*p* = 0.01 and <0.0001, respectively).
(2) In both the unadjusted and adjusted models, the mean HINT-8 scores were significantly higher in the participants who performed ≥500 MET-min/week of leisure-related PA as compared with those who did not (95% CI: 1.017–1.033; *p* < 0.001 and 95% CI: 1.005–1.02; *p* = 0.001, respectively).
(3) In both the unadjusted and adjusted models, the mean HINT-8 scores were significantly higher (95% CI: 1.015–1.03; *p* < 0.001 and 95% CI: 1.004–1.018; *p* = 0.003, respectively) in the participants who performed MSPA for ≥2 days/week as compared with those who did not.

## 5. Strengths and Limitations

The current study has several limitations. First, to date, the relationship between PA and HR QOL has been studied based on self-reported measurements of PA [10]. Self-reported measurements of PA have a poor correlation with direct measurements of it and are affected by several biases, such as recall and social desirability biases [57]. In particular, it is difficult to precisely recall the duration and intensity of PA [57,58,59]. Therefore, the assessment of a dose–response relationship between PA and HR QOL deserves further studies. Second, the current study failed to assess the possible relationship between HR QOL and the time of sedentary behavior or that spent on light-intensity PA. Both sedentary behavior and light-intensity PA are of special interest among older adults and individuals with chronic conditions, both of whom are characterized by less physical activity as compared with the general population and spend most of their active time on light-intensity PA [60,61,62].

## 6. Conclusions

The current results confirmed that there is a significant positive correlation between PA and HR QOL based on the HINT-8 scores. Because the HINT-8 was developed to assess HR QOL in Koreans, however, further studies are warranted to evaluate its applicability to other ethnic populations.

## Figures and Tables

**Table 1:** Questionnaires and levels of the Health-related Quality of Life Instrument with 8 Items.

Domains	Items	Levels of Responses
**Physical health**	**Stair Climbing**	1. I had no difficulty climbing stairs.
		2. I had some difficulty climbing stairs.
		3. I had a lot of difficulty climbing stairs.
		4. I couldn’t climb stairs.
	**Pain**	1. I had no pain.
		2. I had mild pain.
		3. I had severe pain.
		4. I had intense pain.
	**Vitality**	1. I always had energy.
		2. I often had energy.
		3. I sometimes had energy.
		4. I had no energy at all.
**Social health**	**Working**	1. I had no difficulty working.
		2. I had some difficulty working.
		3. I had a lot of difficulty working.
		4. I couldn’t work.
**Mental health**	**Depression**	1. I was not depressed at all.
		2. I was occasionally depressed.
		3. I was frequently depressed.
		4. I was always depressed.
	**Memory**	1. I had no difficulty remembering.
		2. I had some difficulty remembering.
		3. I had a lot of difficulty remembering.
		4. I couldn’t remember at all.
	**Sleep**	1. I had no difficulty sleeping.
		2. I had some difficulty sleeping.
		3. I had a lot of difficulty sleeping.
		4. I couldn’t sleep well.
**Positive health**	**Happiness**	1. I was always happy.
		2. I was often happy.
		3. I was occasionally happy.
		4. I was not happy at all.

**Table 2:** Demographic characteristics of the participants.

		Men (*n* = 1941)	Women (*n* = 2416)	Total (*n* = 4357)
Age (Years Old)		44 (33–55)	46 (35–55)	45 (35–55)
**Age groups (range)**				
	Young adults (19–34 years)	536 (27.6%)	538 (22.3%)	1074 (24.6%)
	Early middle-aged adults (35–49 years)	698 (36%)	892 (36.9%)	1590 (36.5%)
	Late middle-aged adults (50–64 years)	707 (36.4%)	986 (40.8%)	1693 (38.9%)
**Height (cm)**		172.8 (168.6–177)	159.7 (155.5–163.5)	165 (158.9–172.1)
**Weight (kg)**		72.8 (65.8–80.7)	57.5 (52.5–64)	63.8 (55.9–73.9)
**BMI (kg/m^2^)**		24.4 (22.4–26.7)	22.7 (20.6–25.1)	23.5 (21.3–26)
	<23	611 (31.5%)	1327 (54.9%)	1938 (44.5%)
	23–25	487 (25.1%)	463 (19.2%)	950 (21.8%)
	>25	843 (43.4%)	626 (25.9%)	1469 (33.7%)
**Levels of education**				
	Elementary school graduates	68 (3.5%)	174 (7.2%)	242 (5.6%)
	Middle school graduates	125 (6.4%)	181 (7.5%)	306 (7%)
	High school graduates	759 (39.1%)	919 (38%)	1678 (38.5%)
	≥College or university graduates	989 (51%)	1142 (47.3%)	2131 (48.9%)
**Monthly household income (KRW)**				
	Low	165 (8.5%)	238 (9.9%)	403 (9.2%)
	Middle low	452 (23.3%)	609 (25.2%)	1061 (24.4%)
	Middle high	571 (29.4%)	692 (28.6%)	1263 (29%)
	High	753 (38.8%)	877 (36.3%)	1630 (37.4%)
**Economic activities**				
	Yes	1590 (81.9%)	1452 (60.1%)	3042 (69.8%)
	No	351 (18.1%)	964 (39.9%)	1315 (30.2%)
**Marital status**				
	Married	1360 (70.1%)	1985 (82.2%)	3345 (76.8%)
	Others	581 (29.9%)	431 (17.8%)	1012 (23.2%)
**Smoking status**				
	Non-smokers	488 (25.1%)	2068 (85.6%)	2556 (58.7%)
	Ex-smokers	727 (37.5%)	200 (8.3%)	927 (21.3%)
	Smokers	726 (37.4%)	148 (6.1%)	874 (20.1%)
**Alcohol intake**				
	Abstinent	233 (12%)	641 (26.5%)	874 (20.1%)
	Low moderate	479 (24.7%)	926 (38.3%)	1405 (32.2%)
	Occasional	574 (29.6%)	514 (21.3%)	1088 (25%)
	Frequent	655 (33.7%)	335 (13.9%)	990 (22.7%)
**Medical history**				
	Hypertension	316 (16.3%)	297 (12.3%)	613 (14.1%)
	Diabetes mellitus	136 (7%)	105 (4.3%)	241 (5.5%)
	Dyslipidemia	241 (12.4%)	343 (14.2%)	584 (13.4%)
	Cerebrovascular diseases	26 (1.3%)	15 (0.6%)	41 (0.9%)
	Ischemic heart diseases	38 (2%)	11 (0.5%)	49 (1.1%)
**Adherence to PA guidelines**				
	MVPA (≥500 MET-min/week)	516 (26.6%)	346 (14.3%)	862 (19.8%)
	Strengthening (≥2 days/week)	631 (32.5%)	372 (15.4%)	1003 (23%)
**MVPA categories**				
	Inactive	1154 (59.5%)	1782 (73.8%)	2936 (67.4%)
	Insufficiently active	271 (14%)	288 (11.9%)	559 (12.8%)
	Active	203 (10.5%)	166 (6.9%)	369 (8.5%)
	Highly active	313 (16.1%)	180 (7.5%)	493 (11.3%)

Abbreviations: BMI, body mass index; PA, physical activity; MVPA, moderate-to-vigorous-intensity physical activity; MET, metabolic equivalent of task. Values are the number of the participants with percentage or median with interquartile range, where appropriate.

**Table 3:** Relationship between leisure-related physical activity and the Health-Related Quality of Life Instrument with 8 Items scores—unadjusted model.

		Unadjusted Model						
		B	SE	Wald χ^2^	Exp (β)	Percentile Difference (%)	95% CI	*p*-Value
**Leisure-related PA**								
	<500 MET-min/week				Ref.			
	≥500 MET-min/week	0.025	0.0038	41.8	1.025	2.510	(1.017–1.033)	<0.001

Note: SE, standard error; CI, confidence interval. Abbreviations: PA, physical activity; MET, metabolic equivalent of task.

**Table 4:** Relationship between leisure-time physical activity and the Health-Related Quality of Life Instrument with 8 Items scores—adjusted model.

		Adjusted Model						
		B	SE	Wald χ^2^	Exp (β)	Percentile Difference (%)	95% CI	*p*-Value
**Leisure-related PA**								
	<500 MET-min/week				Ref.			
	≥500 MET-min/week	0.013	0.0037	11.5	1.013	1.27	(1.005–1.02)	0.001
**Sex**								
	Men				Ref.			
	Women	−0.039	0.0039	97.6	0.962	−3.82	(0.954–0.969)	<0.001
**Age (years old)**								
	19–34				Ref.			
	35–49	−0.014	0.0048	8.9	0.986	−1.41	(0.977–0.995)	0.003
	50–64	−0.022	0.0053	17.7	0.978	−2.22	(0.968–0.988)	<0.001
**Levels of education**								
	Elementary school graduates				Ref.			
	Middle school graduates	0.017	0.0083	4.0	1.017	1.67	(1–1.033)	0.046
	High school graduates	0.043	0.0070	37.8	1.044	4.37	(1.03–1.058)	<0.001
	≥College or university graduates	0.045	0.0072	38.4	1.046	4.56	(1.031–1.06)	<0.001
**Monthly household income**								
	Low				Ref.			
	Mid-low	0.023	0.0057	16.8	1.024	2.37	(1.012–1.035)	<0.001
	Mid-high	0.035	0.0057	38.5	1.036	3.59	(1.024–1.047)	<0.001
	High	0.035	0.0057	37.6	1.035	3.53	(1.024–1.047)	<0.001
**Economic activities**								
	Yes				Ref.			
	No	−0.016	0.0034	22.6	0.984	−1.59	(0.978–0.991)	<0.001
**Marital status**								
	Married				Ref.			
	Others	−0.013	0.0047	7.0	0.988	−1.25	(0.978–0.997)	0.008
**Smoking status**								
	Non-smokers				Ref.			
	Ex-smokers	−0.013	0.0044	8.6	0.987	−1.27	(0.979–0.996)	0.003
	Smokers	−0.029	0.0046	38.0	0.972	−2.81	(0.963–0.981)	<0.001
**Alcohol intake**								
	Abstinent				Ref.			
	Low-moderate	0.001	0.0042	0.1	1.001	0.14	(0.993–1.01)	0.734
	Occasional	0.009	0.0046	3.9	1.009	0.91	(1–1.018)	0.047
	Frequent	0.005	0.0048	1.1	1.005	0.51	(0.996–1.014)	0.287
**BMI (kg/m^2^)**								
	<23				Ref.			
	23–25	−0.005	0.0039	1.7	0.995	−0.50	(0.988–1.003)	0.196
	>25	−0.009	0.0035	7.2	0.991	−0.93	(0.984–0.998)	0.007
**Medical history**								
	Hypertension	−0.006	0.0048	1.8	0.994	−0.64	(0.984–1.003)	0.181
	Diabetes mellitus	−0.011	0.0068	2.4	0.989	−1.06	(0.976–1.003)	0.120
	Dyslipidemia	−0.017	0.0049	12.5	0.983	−1.71	(0.974–0.992)	<0.001
	Cerebrovascular diseases	−0.049	0.0151	10.6	0.952	−4.81	(0.924–0.981)	0.001
	Ischemic heart diseases	−0.023	0.0140	2.7	0.977	−2.26	(0.951–1.005)	0.101

Note: SE, standard error; CI, confidence interval. Abbreviations: PA, physical activity; MET, metabolic equivalent of task; BMI, body mass index.

**Table 5:** Relationship between muscle-strengthening physical activity with the Health-Related Quality of Life Instrument with 8 Items scores—Unadjusted model.

		Unadjusted Model						
		B	SE	Wald χ^2^	Exp (β)	Percentile Difference (%)	95% CI	*p*-Value
**MSPA**								
	<2 days/week				Ref.			
	≥2 days/week	0.022	0.0036	37.8	1.023	2.26	(1.015–1.03)	<0.001

Note: SE, standard error; CI, confidence interval. Abbreviations: MSPA, muscle-strengthening physical activity.

**Table 6:** Relationship between muscle-strengthening physical activity with the Health-Related Quality of Life Instrument with 8 Items scores—Adjusted model.

		Adjusted Model						
		B	SE	Wald χ^2^	Exp (β)	Percentile Difference (%)	95% CI	*p*-Value
**MSPA**								
	<2 days/week				Ref.			
	≥2 days/week	0.011	0.0035	9.0	1.011	1.06	(1.004–1.018)	0.003
**Sex**								
	Men				Ref.			
	Women	−0.039	0.0040	94.9	0.962	−3.79	(0.955–0.97)	<0.001
**Age (years old)**								
	19–34				Ref.			
	35–49	−0.014	0.0048	8.8	0.986	−1.40	(0.977–0.995)	0.003
	50–64	−0.023	0.0053	18.6	0.977	−2.28	(0.967–0.987)	<0.001
**Levels of education**								
	Elementary school graduates				Ref.			
	Middle school graduates	0.016	0.0083	3.5	1.016	1.57	(0.999–1.032)	0.060
	High school graduates	0.042	0.0070	36.9	1.043	4.32	(1.029–1.058)	<0.001
	≥College or university graduates	0.044	0.0072	37.6	1.045	4.51	(1.03–1.06)	<0.001
**Monthly household income**								
	Low				Ref.			
	Mid-low	0.023	0.0057	16.6	1.024	2.35	(1.012–1.035)	<0.001
	Mid-high	0.035	0.0057	39.0	1.036	3.61	(1.025–1.048)	<0.001
	High	0.035	0.0057	38.8	1.036	3.58	(1.024–1.047)	<0.001
**Economic activities**								
	Yes				Ref.			
	No	−0.016	0.0034	21.9	0.984	−1.56	(0.978–0.991)	<0.001
**Marital status**								
	Married				Ref.			
	Others	−0.013	0.0047	7.2	0.987	−1.26	(0.978–0.997)	0.007
**Smoking status**								
	Non-smokers				Ref.			
	Ex-smokers	−0.013	0.0044	8.6	0.987	−1.27	(0.979–0.996)	0.003
	Smokers	−0.029	0.0046	38.7	0.972	−2.83	(0.963–0.981)	<0.001
**Alcohol intake**								
	Abstinent				Ref.			
	Low-moderate	0.002	0.0042	0.2	1.002	0.17	(0.994–1.01)	0.683
	Occasional	0.009	0.0046	4.2	1.009	0.93	(1–1.018)	0.042
	Frequent	0.005	0.0048	1.2	1.005	0.53	(0.996–1.015)	0.265
**BMI (kg/m^2^)**								
	<23				Ref.			
	23–25	−0.005	0.0039	1.5	0.995	−0.47	(0.988–1.003)	0.218
	>25	−0.009	0.0035	6.5	0.991	−0.88	(0.984–0.998)	0.011
**Medical history**								
	Hypertension	−0.007	0.0048	1.9	0.993	−0.65	(0.984–1.003)	0.169
	Diabetes mellitus	−0.010	0.0068	2.2	0.990	−1.00	(0.977–1.003)	0.141
	Dyslipidemia	−0.017	0.0049	12.3	0.983	−1.70	(0.974–0.992)	<0.001
	Cerebrovascular diseases	−0.050	0.0151	10.8	0.952	−4.84	(0.924–0.98)	0.001
	Ischemic heart diseases	−0.021	0.0140	2.3	0.979	−2.12	(0.952–1.006)	0.127

Note: SE, standard error; CI, confidence interval. Abbreviations: MSPA, muscle-strengthening physical activity; BMI, body mass index.

## Data Availability

The KNHANES database is publicly available (https://knhanes.kdca.go.kr (accessed on 28 May 2023) [in Korean]).

## References

[1] Marquez D.X., Aguiñaga S., Vásquez P.M., Conroy D.E., Erickson K.I., Hillman C., Stillman C.M., Ballard R.M., Sheppard B.B., Petruzzello S.J. (2020). A systematic review of physical activity and quality of life and well-being. Transl. Behav. Med..

[2] Park B.Y., Ko D.S., Park H.S. (2013). Relationship between job characteristic and quality of life among some elderly. J. Korean Inst. Electronic. Commun. Sci..

[3] Kim D.J. (2019). The Effects of Restricted Physical Activity on Health-Related Quality of Life in Adult Patients with Depression. Osong Public Health Res. Perspect..

[4] Antunes H.K., Stella S.G., Santos R.F., Bueno O.F., de Mello M.T. (2005). Depression, anxiety and quality of life scores in seniors after an endurance exercise program. Braz. J. Psychiatry.

[5] Morgan W.P., O’Connor P.J., Ellickson K.A., Bradley P.W. (1988). Personality structure, mood states, and performance in elite male distance runners. Int. J. Sport Psychol..

[6] Awick E.A., Ehlers D.K., Aguiñaga S., Daugherty A.M., Kramer A.F., McAuley E. (2017). Effects of a randomized exercise trial on physical activity, psychological distress and quality of life in older adults. Gen. Hosp. Psychiatry.

[7] Fabre C., Massé-Biron J., Chamari K., Varray A., Mucci P., Préfaut C. (1999). Evaluation of quality of life in elderly healthy subjects after aerobic and/or mental training. Arch. Gerontol. Geriatr..

[8] Joos B., Uebelhart D., Michel B.A., Sprott H. (2004). Influence of an outpatient multidisciplinary pain management program on the health-related quality of life and the physical fitness of chronic pain patients. J. Negat. Results Biomed..

[9] Oldervoll L.M., Kaasa S., Hjermstad M.J., Lund J.A., Loge J.H. (2004). Physical exercise results in the improved subjective well-being of a few or is effective rehabilitation for all cancer patients?. Eur. J. Cancer.

[10] Bize R., Johnson J.A., Plotnikoff R.C. (2007). Physical activity level and health-related quality of life in the general adult population: A systematic review. Prev. Med..

[11] Anokye N.K., Trueman P., Green C., Pavey T.G., Taylor R.S. (2012). Physical activity and health related quality of life. BMC Public Health.

[12] Blom E.E., Aadland E., Skrove G.K., Solbraa A.K., Oldervoll L.M. (2019). Health-related quality of life and intensity-specific physical activity in high-risk adults attending a behavior change service within primary care. PLoS ONE.

[13] Kokic I.S., Znika M., Brumnic V. (2019). Physical activity, health-related quality of life and musculoskeletal pain among students of physiotherapy and social sciences in Eastern Croatia—Cross-sectional survey. Ann. Agric. Environ. Med..

[14] Wafa S.W., Shahril M.R., Ahmad A.B., Zainuddin L.R., Ismail K.F., Aung M.M., Mohd Yusoff N.A. (2016). Association between physical activity and health-related quality of life in children: A cross-sectional study. Health Qual. Life Outcomes.

[15] Psarrou A., Adamakidou T., Apostolara P., Koreli A., Drakopoulou M., Plakas S., Mastrogiannis D., Mantoudi A., Parissopoulos S., Zartaloudi A. (2023). Associations between Physical Activity and Health-Related Quality of Life among Community-Dwelling Older Adults: A Cross-Sectional Study in Urban Greece. Geriatrics.

[16] Mendes M.A., da Silva I., Ramires V., Reichert F., Martins R., Ferreira R., Tomasi E. (2018). Metabolic equivalent of task (METs) thresholds as an indicator of physical activity intensity. PLoS ONE.

[17] Nikitas C., Kikidis D., Bibas A., Pavlou M., Zachou Z., Bamiou D.E. (2022). Recommendations for physical activity in the elderly population: A scoping review of guidelines. J. Frailty Sarcopenia Falls.

[18] Lee M.N., Kim S.D., Choi Y.S. (2022). The Relationship between Physical Activity and Health-Related Quality of Life (HINT-Eight) in Middle-Aged Korean Women. J. Environ. Public Health.

[19] Kim W., Han K.T., Kim S. (2022). Health-related quality of life among cancer patients and survivors and its relationship with current employment status. Support. Care Cancer.

[20] Seo M.H., Lee W.Y., Kim S.S., Kang J.H., Kang J.H., Kim K.K., Kim B.Y., Kim Y.H., Kim W.J., Kim E.M. (2019). 2018 Korean Society for the Study of Obesity guideline for the management of obesity in Korea. J. Obes. Metab. Syndr..

[21] Kim N., Kim G.-U., Kim H. (2020). Comparative Study of Dietary Patterns by Living Arrangements: The Korea National Health and Nutrition Examination Survey (KNHANES) 2013–2015. Int. J. Environ. Res. Public Health.

[22] GBD 2017 Mortality Collaborators (2018). Global, regional, and national age-sex-specific mortality and life expectancy, 1950–2017: A systematic analysis for the Global Burden of Disease Study 2017. Lancet.

[23] GBD 2017 Disease and Injury Incidence and Prevalence Collaborators (2018). Global, regional, and national incidence, prevalence, and years lived with disability for 354 diseases and injuries for 195 countries and territories, 1990–2017: A systematic analysis for the Global Burden of Disease Study 2017. Lancet.

[24] GBD 2017 Risk Factor Collaborators (2018). Global, regional, and national comparative risk assessment of 84 behavioural, environmental and occupational, and metabolic risks or clusters of risks for 195 countries and territories, 1990–2017: A systematic analysis for the Global Burden of Disease Study 2017. Lancet.

[25] Pedersen B.K., Saltin B. (2015). Exercise as medicine—Evidence for prescribing exercise as therapy in 26 different chronic diseases. Scand. J. Med. Sci. Sports.

[26] DiPietro L., Buchner D.M., Marquez D.X., Pate R.R., Pescatello L.S., Whitt-Glover M.C. (2019). New scientific basis for the 2018 U.S. Physical Activity Guidelines. J. Sport Health Sci..

[27] Rejeski W.J., Mihalko S.L. (2001). Physical activity and quality of life in older adults. J. Gerontol. A Biol. Sci. Med. Sci..

[28] Halaweh H., Willen C., Grimby-Ekman A., Svantesson U. (2015). Physical Activity and Health-Related Quality of Life Among Community Dwelling Elderly. J. Clin. Med. Res..

[29] Cho K.O. (2014). The Positive Effect of Physical Activity on Health and Health-related Quality of Life in Elderly Korean People-Evidence from the Fifth Korea National Health and Nutrition Examination Survey. J. Lifestyle Med..

[30] Eckert K. (2012). Impact of physical activity and bodyweight on health-related quality of life in people with type 2 diabetes. Diabetes Metab. Syndr. Obes..

[31] Gillison F.B., Skevington S.M., Sato A., Standage M., Evangelidou S. (2009). The effects of exercise interventions on quality of life in clinical and healthy populations; a meta-analysis. Soc. Sci. Med..

[32] Floegel T.A., Perez G.A. (2016). An integrative review of physical activity/exercise intervention effects on function and health-related quality of life in older adults with heart failure. Geriatr. Nurs..

[33] Eaglehouse Y.L., Schafer G.L., Arena V.C., Kramer M.K., Miller R.G., Kriska A.M. (2016). Impact of a community-based lifestyle intervention program on health-related quality of life. Qual. Life. Res..

[34] Scarabottolo C.C., Cyrino E.S., Nakamura P.M., Tebar W.R., da Canhin D.S., Gobbo L.A., Christofaro D.G.D. (2019). Relationship of Different Domains of Physical Activity Practice with Health-Related Quality of Life among Community-Dwelling Older People: A Cross-Sectional Study. BMJ Open.

[35] Bădicu G. (2018). Physical Activity and Health-Related Quality of Life in Adults from Braşov, Romania. Educ. Sci..

[36] Subramaniam M., Zhang Y., Lau J.H., Vaingankar J.A., Abdin E., Chong S.A., Lee E.S. (2019). Patterns of Physical Activity and Health-Related Quality of Life amongst Patients with Multimorbidity in a Multi-Ethnic Asian Population. BMC Public Health.

[37] (1995). The World Health Organization Quality of Life assessment (WHOQOL): Position paper from the World Health Organization. Soc. Sci. Med..

[38] Schwimmer J.B., Burwinkle T.M., Varni J.W. (2003). Health-related quality of life of severely obese children and adolescents. JAMA.

[39] Jayasinghe U.W., Harris M.F., Parker S.M., Litt J., van Driel M., Mazza D., Del Mar C., Lloyd J., Smith J., Zwar N. (2016). Preventive Evidence into Practice (PEP) Partnership Group. The impact of health literacy and life style risk factors on health-related quality of life of Australian patients. Health Qual. Life Outcomes.

[40] Fone D., Dunstan F., Lloyd K., Williams G., Watkins J., Palmer S. (2007). Does social cohesion modify the association between area income deprivation and mental health? A multilevel analysis. Int. J. Epidemiol..

[41] Keles H., Ekici A., Ekici M., Bulcun E., Altinkaya V. (2007). Effect of chronic diseases and associated psychological distress on health-related quality of life. Intern. Med. J..

[42] Ruiz-Comellas A., Valmaña G.S., Catalina Q.M., Baena I.G., Peña J.M., Poch P.R., Carrera A.S., Pujol I.C., Solà C., Gamisans M.F. (2022). Effects of Physical Activity Interventions in the Elderly with Anxiety, Depression, and Low Social Support: A Clinical Multicentre Randomised Trial. Healthcare.

[43] Fishwick D., Lewis L., Darby A., Young C., Wiggans R., Waterhouse J., Wight J., Blanc P.D. (2015). Determinants of health-related quality of life among residents with and without COPD in a historically industrialised area. Int. Arch. Occup. Environ. Health.

[44] Tan Z., Liang Y., Liu S., Cao W., Tu H., Guo L., Xu Y. (2013). Health-related quality of life as measured with EQ-5D among populations with and without specific chronic conditions: A population-based survey in Shaanxi Province, China. PLoS ONE.

[45] Jang E.S., Kim Y.S., Kim K.A., Lee Y.J., Chung W.J., Kim I.H., Lee B.S., Jeong S.H. (2018). Factors Associated with Health-Related Quality of Life in Korean Patients with Chronic Hepatitis C Infection Using the SF-36 and EQ-5D. Gut Liver.

[46] Liu L., Li S., Wang M., Chen G. (2017). Comparison of EQ-5D-5L health state utilities using four country-specific tariffs on a breast cancer patient sample in mainland China. Patient Prefer. Adherence.

[47] Kim S., Kwon Y.M., Park Y.I. (2014). Association between Physical Activity and Health-Related Quality of Life in Korean: The Korea National Health and Nutrition Examination Survey IV. Korean J. Fam. Med..

[48] Son M., Sung H., Kim Y. (2021). The Association between Resistance Exercise Frequency, Aerobic Physical Activity Level, and Health-Related Quality of Life in Korean Older Adults: Findings from the Seventh Korea National Health and Nutrition Examination Survey, 2018. Korean J. Sports Med..

[49] Lee S.N., Lee H.S., Lee S.W., Shim K.W., Song G.Y., Byun A.R. (2020). Association between Physical Activity and Health-Related Quality of Life in Korean Patients with Diabetes Mellitus. Korean J. Fam. Pract..

[50] Ryu M., Lee S., Kim H., Baek W.-C., Kimm H. (2020). Effect of Aerobic Physical Activity on Health-Related Quality of Life in Middle Aged Women with Osteoarthritis: Korea National Health and Nutrition Examination Survey (2016–2017). Int. J. Environ. Res. Public Health.

[51] Kong K.A., Kim Y.E., Lim S., Kim B.Y., Kim G.E., Kim S.I. (2022). Depressive Symptoms and Suicidal Ideation in Individuals Living Alone in South Korea. Diagnostics.

[52] Kim Y.-R. (2022). Mediating Effect of Self-Cognitive Oral Health Status on the Effect of Obstructive Sleep Apnea Risk Factors on Quality of Life (HINT-8) in Middle-Aged Korean Women: The Korea National Health and Nutrition Examination Survey. Life.

[53] Lee J.E., Ahn J.H. (2019). A study on deriving a conversion formulae using mapping between HINT-8 and EQ-5D instruments. Korean J. Health Econ. Policy.

[54] Seo J., An S., Kim D. (2023). Effect of Physical Activity on Health-Related Quality of Life of Older Adults Using Newly Developed Health-Related Quality of Life Tool for the Korean Population. Healthcare.

[55] Jang K.-A., Kim Y.-R. (2023). Effects of Muscular Strength Training on Oral Health and Quality of Life: Using Korean Panel Survey Data, a Cross-Sectional Study. Healthcare.

[56] Global Recommendations on Physical Activity for Health. https://www.who.int/publications/i/item/9789241599979.

[57] Warren J.M., Ekelund U., Besson H., Mezzani A., Geladas N., Vanhees L. (2010). Assessment of physical activity—A review of methodologies with reference to epidemiological research: A report of the exercise physiology section of the European Association of Cardiovascular Prevention and Rehabilitation. Eur. J. Cardiovasc. Prev. Rehabil..

[58] Sallis J.F., Saelens B.E. (2000). Assessment of physical activity by self-report: Status, limitations, and future directions. Res. Q. Exerc. Sport..

[59] Prince S.A., Adamo K.B., Hamel M.E., Hardt J., Gorber S.C. (2008). A comparison of direct versus self-report measures for assessing physical activity in adults: A systematic review. Int. J. Behav. Nutr. Phys. Act..

[60] Ashe M.C., Miller W.C., Eng J.J., Noreau L., Physical Activity and Chronic Conditions Research Team (2009). Older adults, chronic disease and leisure-time physical activity. Gerontology.

[61] Keats M.R., Cui Y., DeClercq V., Dummer T.J.B., Forbes C., Grandy S.A., Hicks J., Sweeney E., Yu Z.M., Parker L. (2017). Multimorbidity in Atlantic Canada and association with low levels of physical activity. Prev. Med..

[62] Buman M.P., Hekler E.B., Haskell W.L., Pruitt L., Conway T.L., Cain K.L., Sallis J.F., Saelens B.E., Frank L.D., King A.C. (2010). Objective light-intensity physical activity associations with rated health in older adults. Am. J. Epidemiol..

